# Long-Term Premorbid Blood Pressure and Cerebral Small Vessel Disease Burden on Imaging in Transient Ischemic Attack and Ischemic Stroke: Population-Based Study

**DOI:** 10.1161/STROKEAHA.118.021578

**Published:** 2018-07-25

**Authors:** Kui Kai Lau, Linxin Li, Michela Simoni, Ziyah Mehta, Wilhelm Küker, Peter M. Rothwell

**Affiliations:** From the Centre for Prevention of Stroke and Dementia, Nuffield Department of Clinical Neurosciences, University of Oxford, United Kingdom.

**Keywords:** blood pressure, magnetic resonance imaging, neuroimaging, stroke

## Abstract

**Background and Purpose—:**

Studies of causes of cerebral small vessel disease (SVD) should fully adjust for blood pressure (BP), but most etiological studies use a single BP measurement or history of hypertension, which might underestimate the role of hypertension. In patients with transient ischemic attack and ischemic stroke, we therefore compared the associations of baseline and long-term premorbid BP with measures of SVD on magnetic resonance imaging brain.

**Methods—:**

We studied 1009 transient ischemic attack/ischemic stroke patients who had a brain magnetic resonance imaging, in the population-based OXVASC (Oxford Vascular Study), and related baseline and 20-year premorbid BP (median: 15 readings/patient) to the total SVD score on imaging.

**Results—:**

SVD score was associated with increasing mean baseline systolic BP (SBP; odds ratio of top versus bottom BP quartile: 2.28; [95% CI, 1.62–3.21]; *P*<0.0001) and with prior hypertension (2.53; [95% CI, 2.01–3.20]; *P*<0.0001), but the association was much stronger with mean premorbid SBP (6.09; [95% CI, 4.34–8.55]; *P*<0.0001). Mean diastolic BP at baseline was negatively associated with SVD score (0.71; [95% CI, 0.51–1.00]; *P*=0.050), and a positive association was only evident for diastolic BP 10 to 20 years previously (3.35; [95% CI, 2.33–4.84]; both *P*<0.0001). Relationships between overall mean premorbid BP and SVD burden were strongest in patients age <70 (SBP: 6.99; 4.11–11.86; diastolic BP: 3.13; 1.95–5.07; both *P*<0.0001) versus ≥70 years (2.37; 1.42–3.94; *P*=0.001; and 1.16; 0.74–1.84; *P*=0.52).

**Conclusions—:**

Mean premorbid SBP is more strongly associated with SVD burden than baseline SBP or history of hypertension, and baseline diastolic BP yields a misleading estimate of the likely etiological importance of midlife hypertension for the subsequent development of SVD. Studies of novel potential etiological factors for SVD should aim to adjust for long-term prior BP, and trials of BP lowering with only a few years of follow-up may underestimate the overall impact on SVD.

## Abstract 2

Supplemental Digital Content is available in the text.

Cerebral small vessel disease (SVD), which accounts for 20% to 25% of all strokes and up to 45% of all dementias,^1^ is a slowly progressive disorder, often with subtle features initially,^2^ but frequently progressing into a chronic disabling vasculopathy with cognitive impairment, depression, and gait disturbances.^1,3^ Although the pathogenesis of SVD is incompletely understood,^1^ and novel mechanisms have been postulated,^1,4^ hypertension is one of the leading causes,^1,3,5^ and has been associated with all main neuroimaging biomarkers of SVD—lacunes,^6^ white matter hyperintensity (WMH),^7^ microbleeds,^8^ and magnetic resonance imaging (MRI)-visible enlarged perivascular spaces (PVSs),^9^ as well as the global burden of SVD as assessed by the total SVD score.^10^

The majority of previous studies on the association between hypertension and SVD (or other novel risk factors where hypertension has been adjusted for) have been cross-sectional, or have based on single clinic or ambulatory blood pressure (BP) measurements, known history of hypertension and prior use of antihypertensive agents,^6,7,9,10^ potentially underestimating the effects of BP during the many years before clinical presentation. Since systolic BP (SBP) increases, and diastolic BP (DBP) decreases with age,^11^ and in view of the evidence that midlife hypertension may be an important determinant of later cerebrovascular disease and dementia,^12,13^ it may well be important to consider BP many years before the assessment of SVD. Indeed, in one recent prospective cohort study,^14^ the associations between both SBP and DBP and WMH were attenuated during 3-year follow-up as the mean age of the cohort increased from 70 to 73 years. We showed previously that recent premorbid BP may be a trigger for acute lacunar events^15^ and intracerebral hemorrhage,^16^ but analyses of much longer-term premorbid BP control may be required to reliably determine the association with chronic SVD, which is a slowly progressive disorder. To better understand the role of long-term prior BP in development of SVD before transient ischemic attack (TIA)/ischemic stroke, we determined the age-specific time-course of premorbid BP in relation to the total SVD score^17^ in the population-based OXVASC (Oxford Vascular Study).

## Methods

Request for access to data will be considered by the corresponding author.

We prospectively studied patients with TIA/ischemic stroke from OXVASC. In brief, OXVASC is an ongoing population-based study of all acute vascular events occurring within a population of 92 728 individuals, irrespective of age, who are registered with 100 general practitioners in 9 general practices of Oxfordshire, United Kingdom.^18^ The analysis herein includes 1080 consecutive cases of TIA/ischemic stroke recruited from November 1, 2004, to September 30, 2014, who had an MRI brain imaging. The imaging protocol of OXVASC has been described in detail elsewhere.^19,20^ Briefly, from April 1, 2002, to March 31, 2010 (phase 1), MRI and magnetic resonance angiography was performed in selected patients when clinically indicated. From April 1, 2010 onwards (phase 2), brain MRI and magnetic resonance angiography became the first-line imaging methods.

We collected demographic data, atherosclerotic risk factors, details of hospitalization of index event during face-to-face interview and cross-referenced these with primary care and hospital records. All patients had their BPs measured during ascertainment using an oscillometric BP measurement device (A&D Medical, Japan). BPs were taken after 5 minutes of rest in the sitting or lying position, and a single BP reading was used for analysis. Hypertension was defined as known history of hypertension or prior use of antihypertensive agents. We also collected premorbid BP readings from the primary care records (both paper and electronic) for all patients during the preceding 20 years before ascertainment and calculated the mean of all readings, and readings taken between 1 to 5 years, 5 to 10 years, and 10 to 20 years before TIA/ischemic stroke were used for analysis.

Patients were scanned predominantly (856 out of 1009 patients) with either of 2 scanners—Achieva, Philips Healthcare (1.5T, n=481), and Magnetom Verio, Siemens Healthcare (3T, n=375).^19,20^ Details of scan parameters are provided in Table I in the online-only Data Supplement. MRI-visible enlarged PVSs were defined as small (<3 mm) punctate (if perpendicular to the plane of scan) or linear (if longitudinal to the plane of scan) hyperintensities on T2 images in the basal ganglia based on a previously validated scale.^21^ Burden of PVSs was then stratified into 3 groups: <11, 11 to 20, and >20. The severity of WMH was determined for each patient according to the Fazekas scale.^22^ Cerebral microbleeds were defined as rounded, hypodense foci up to 10 mm in size and were differentiated from microbleed mimics based on current guidelines.^23^ The location and number of microbleeds were scored according to the Microbleed Anatomical Rating Scale.^24^ Lacunes were defined as rounded or ovoid lesions, >3 and <20 mm in diameter, in the basal ganglia, internal capsule, centrum semiovale, or brain stem, of cerebrospinal fluid signal density on T2 and fluid-attenuated inversion recovery and no increased signal on diffusion-weighted imaging.^25^ The total burden of SVD was represented by calculating the total SVD score where one point is allocated to each of the following: (1) presence of lacunes, (2) presence of microbleeds, (3) moderate-severe (>10) MRI-visible enlarged basal ganglia-PVSs, and (4) severe periventricular and moderate-severe deep WMH.^17^

One senior neuroradiologist (Dr Küker), who was blinded to the premorbid and baseline BP readings, provided ongoing supervision of interpretation of the MRI images throughout the study period. Definitions of neuroimaging biomarkers were based on Standards for Reporting Vascular Changes on Neuroimaging (STRIVE).^25^ The intrarater κ for 50 randomly selected scans was: lacunes=0.85, microbleed burden (0, 1, 2–4, ≥5)=0.88, periventricular WMH burden (Fazekas grade 0, 1, 2, 3)=0.66, subcortical WMH burden (Fazekas grade 0, 1, 2, 3)=0.75, and basal ganglia-PVS burden (<11, 11–20, >20)=0.86.

Patients gave written informed consent after an event or assent was obtained from relatives for patients who were unable to provide consent. The study was approved by the local research ethics committee.

### Statistical Analysis

We determined by binary and ordinal logistic regression, the relationships of hypertension, baseline BP (top versus bottom quartile as referent) and mean premorbid BP (top versus bottom quartile) with the presence of lacunes and the total SVD score, in univariate analysis and analyses adjusted for age and sex. Test of parallel lines was performed to examine the equal slope assumption on ordinal logistic regression. We also determined by ordinal logistic regression, the relationships of mean premorbid BP taken within 1 year, 1 to 5 years, 5 to 10 years, and 10 to 20 years before TIA/ischemic stroke with the total SVD score overall, and also stratified analysis by age (<70 versus ≥70—chosen as the approximate median age) and by premorbid use of antihypertensive agents. Given the interrelation between years before event and age at the time of BP measurement, we also determined the relationships of total SVD score with mean premorbid BP of measurements taken when patients were aged ≤55, 56 to 65, 66 to 75, and >75. All analyses were done with SPSS version 22.

### Role of the Funding Source

The funding source had no role in study design, data collection, data analysis, data interpretation, or writing of the report. The corresponding author had full access to all the data in the study and had the final responsibility for the decision to submit for publication.

## Results

One thousand eighty patients were recruited during the study period. After excluding 71 patients (6.6%) with missing clinical, premorbid BP, or imaging data, 1009 patients (TIA, n=528; ischemic stroke, n=481) were included in the final analysis. Details of baseline clinical and imaging characteristics are shown in Table 1. The mean (SD) age of the study population was 68.6 (13.8) years and 52% were male. Fifty-five percent of the study population had a history of hypertension or were on antihypertensive agents. A total of 22 096 premorbid BP readings (median, 15 readings/patient; interquartile range [7–33]; 9 [4–21] in age <70 and 23 [12–41] in aged ≥70) were obtained. The mean (SD) premorbid BP was 139 (14)/80 (8) mm Hg, whereas the mean BP on assessment was 150 (24)/84 (13) mm Hg. The mean (SD) total SVD score was 1.12 (1.11). Compared with patients ≥70 years, those aged <70 were more likely to be men, smokers, had fewer vascular risk factors, better renal function, lower premorbid and baseline mean SBP, and higher premorbid and baseline mean DBP (Table 1). The overall prevalence of individual neuroimaging markers and burden of SVD was also lower in patients aged <70 (Table 1).

**Table 1.**
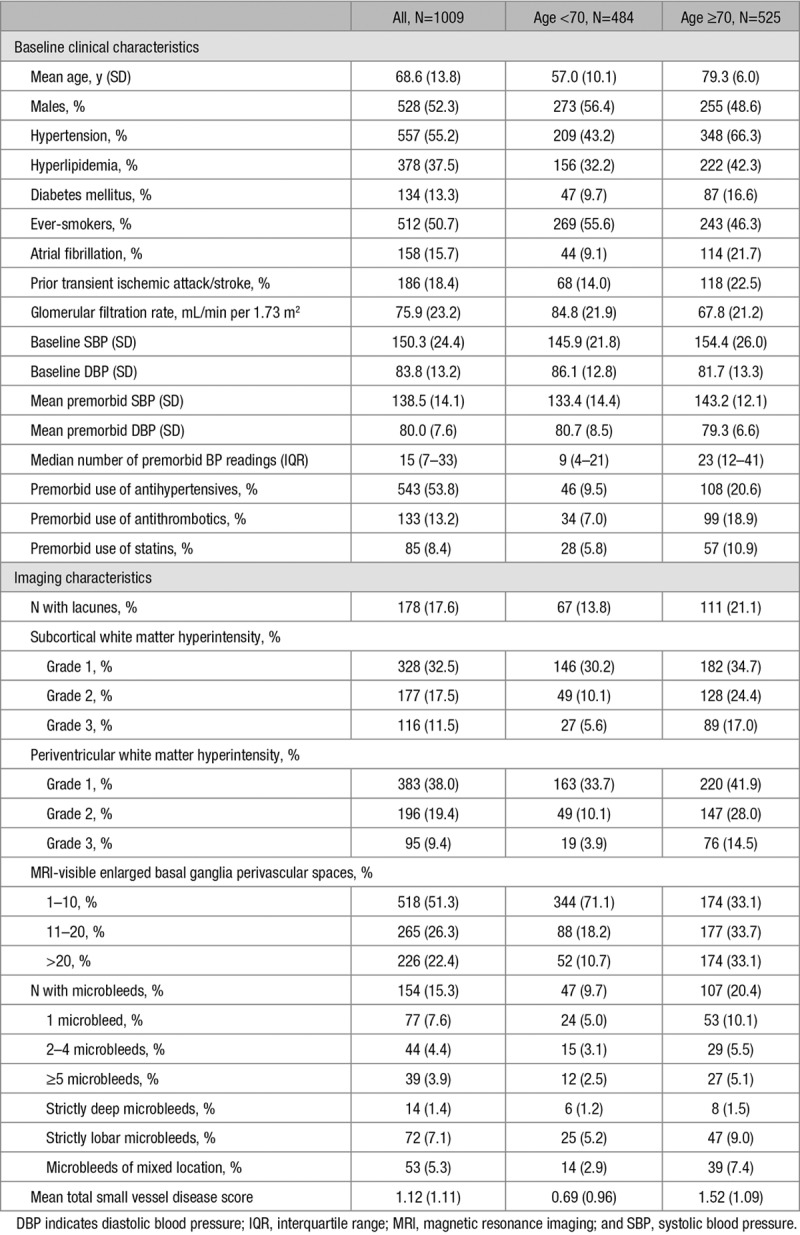
Baseline Clinical and Neuroimaging Characteristics of the Study Population

The relationships of lacunes with baseline BP, history of hypertension, and premorbid BP are shown in Table 2. History of hypertension was significantly associated with the presence of lacunes (age and sex-adjusted odds ratio, 1.74; 95% CI, 1.22–2.49; *P*=0.002), but there were no relationships between baseline SBP (age and sex-adjusted odds ratio of top versus bottom quartile, 1.52; 0.91–2.53; *P*=0.11) or DBP (0.67; 0.40–1.12; *P*=0.12) with lacunes after adjusting for age and sex. However, the associations of mean premorbid SBP and DBP with lacunes was stronger (SBP: 2.92; 1.69–5.03; *P*=0.0001; DBP: 1.99; 1.26–3.16; *P*=0.003). Similar findings were noted for the relationships of baseline and premorbid BP with total SVD score (Table 2). The associations of baseline BP and hypertension with total SVD score were weaker (SBP: 1.46; 1.02–2.10; *P*=0.039; DBP: 1.16; 1.20–1.89; *P*=0.43; hypertension: 1.61; 1.26–2.06; *P*=0.0001) than those of mean premorbid BP (SBP: 2.53; 1.76–3.65; *P*<0.0001; DBP: 2.00; 1.42–2.80; *P*<0.0001; Table 2). The relationships of lacunes and total SVD score with baseline and premorbid BP (per SD increase) is shown in Table II in the online-only Data Supplement.

**Table 2.**
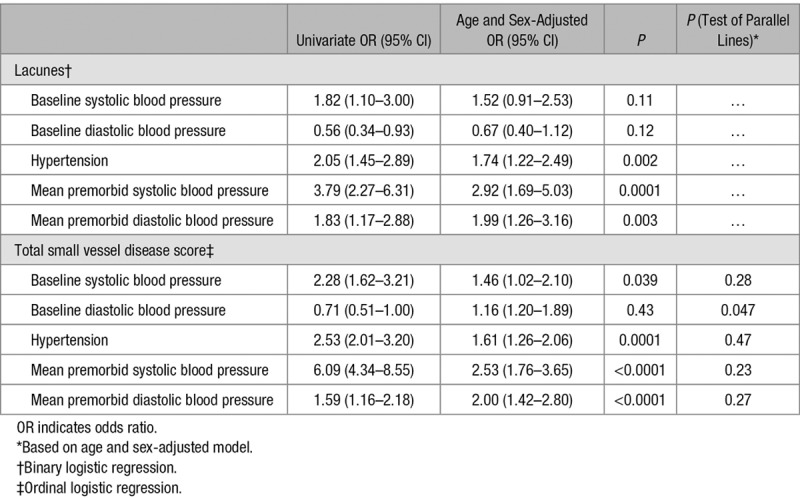
Relationships of Lacunes and Total Small Vessel Disease Score With Baseline Blood Pressure, Premorbid Blood Pressure (Top Versus Bottom Quartile), and History of Hypertension

We determined the relationships between premorbid BP and total SVD score taken at different time points (within 1, 1–5, 5–10, and 10–20 years) before index TIA/ischemic stroke (Table 3; Figure). A clear stepwise increase in strength of the association based on premorbid BP taken within 1 year, 1 to 5 years, 5 to 10 years, and 10 to 20 years was noted in univariate analysis for SBP (2.17, 1.48–3.17; 3.94, 2.78–5.56; 4.67, 3.23–6.76; and 5.92, 4.05–8.65; respectively) and for DBP (0.91, 0.62–1.33; 0.76, 0.54–1.06; 1.26, 0.89–1.79; 3.35, 2.33–4.84; Table 3; Figure). These associations were similar in the 466 (46%) antihypertensive naive patients in univariate analysis (Table 4) but were attenuated after adjusting for age and sex (Tables 3 and 4).

**Table 3.**
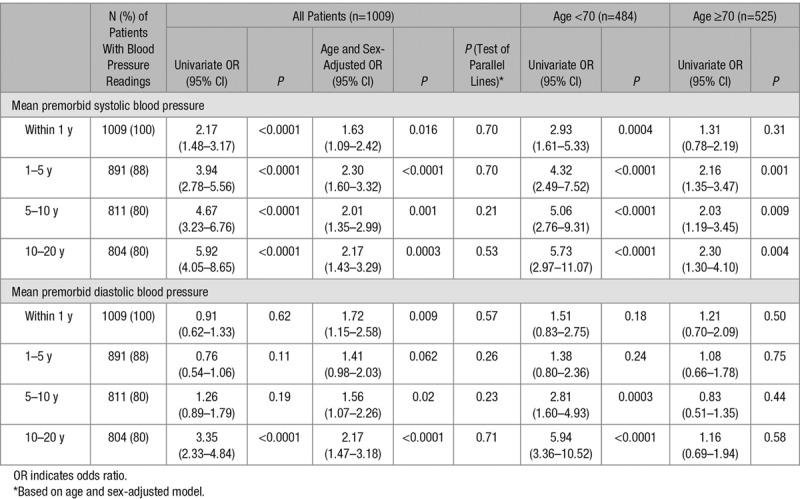
Relationships of Total Small Vessel Disease Score With Premorbid Blood Pressures Measured (Top Versus Bottom Quartile) Within 1 Year, 1 to 5 Years, 5 to 10 Years, and 10 to 20 Years of Transient Ischemic Attack/Ischemic Stroke Using Ordinal Regression

**Table 4.**
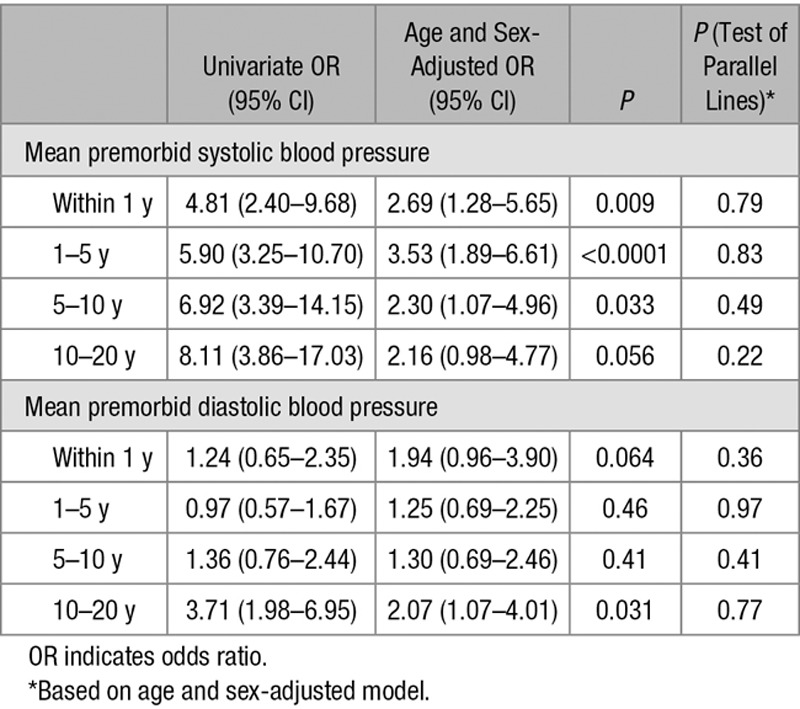
Sensitivity Analysis (Using Ordinal Regression) of the Relationship of Total Small Vessel Disease Score With Mean Premorbid Blood Pressure (Top Versus Bottom Quartile) Excluding Antihypertensive Users

**Figure.**
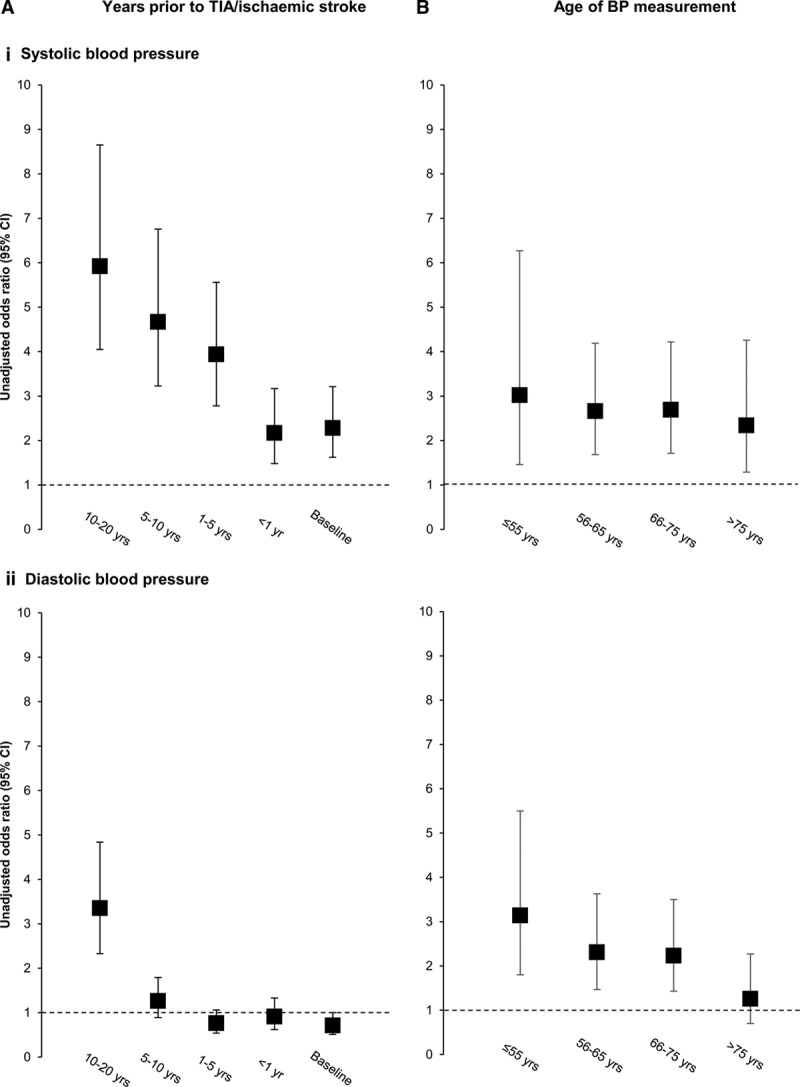
Unadjusted odds ratios for an increasing total small vessel disease score with baseline and mean premorbid (i) systolic and (ii) diastolic blood pressure (BP) stratified by (**A**) years before transient ischemic attack (TIA)/ischemic stroke when BP was measured and (**B**) age of patients when blood pressure was measured (odds ratios of baseline and premorbid BP taken as top vs bottom quartile as referent).

When stratified by age, the associations between premorbid SBP taken at different time points with increasing total SVD score were stronger in the 484 (48%) patients aged <70 compared with those aged ≥70 (Table 3). The associations between mean premorbid DBP and total SVD score were particularly weak at age ≥70 years, even when based on measurements made 10 to 20 years previously (1.16; 0.69–1.94; *P*=0.58; Table 3), in contrast to patients aged <70 (10–20 years: 5.94; 3.36–10.52; *P*<0.0001). In the 251 (25%) patients aged <60 years (Table III in the online-only Data Supplement), the risk association for 10 to 20-year premorbid mean DBP was stronger still (6.56; 2.47–17.41; *P*=0.0002) and exceeded that for mean SBP during the same period (3.65; 0.98–13.61; *P*=0.054).

The importance of age at BP measurement was less evident for risk associations with SVD based on SBP (unadjusted odds ratio of top versus bottom quartile of SVD score of ≤55 years: 3.02, 1.46–6.27; 56–65 years: 2.66, 1.68–4.19; 66–75 years: 2.69, 1.71–4.22; >75 years: 2.34, 1.29–4.26; Table IV in the online-only Data Supplement; Figure), but a clear stepwise decline in strength of association was seen for premorbid DBP with increasing age (≤55 years: 3.14, 1.80–5.50; 56–65 years: 2.31, 1.47–3.63; 66–75 years: 2.23, 1.43–3.50; >75 years: 1.26, 0.70–2.27; Table IV in the online-only Data Supplement; Figure).

## Discussion

We have demonstrated in a large population-based study of TIA/ischemic stroke patients, the different relationships of baseline BP, history of hypertension, and long-term mean premorbid BP with global SVD burden. We found weaker associations for baseline BP and a history of hypertension than for premorbid BP. A latency effect between BP and SVD burden was also present, such that the risk associations with SVD burden were stronger with BP readings taken within more distant time periods before the TIA/ischemic stroke. Furthermore, we demonstrated significant age-specific associations between premorbid BP and SVD burden, with stronger associations, especially DBP, in younger individuals.

Our findings have several implications. First, our results suggest that the importance of hypertension as a risk factor toward SVD is likely to have been underestimated in cross-sectional studies based only on baseline BP or diagnosis of hypertension (often defined as known history of hypertension or on antihypertensive agents).^6,7,9^ As we have demonstrated, multiple premorbid BP readings (median of 15 readings per patient over a 20 year period in our cohort) correlated more strongly with global SVD burden than a single BP measurement or a known history of hypertension. It may well be possible that a proportion of previously noted cases of nonhypertensive cerebral SVD^26^ had either masked hypertension, and a diagnosis of hypertension might have been made with repeated measurements of BP, or have had hypertension in the past. Our findings therefore suggest that studies investigating etiological factors in SVD should ideally aim to adjust for repeated measurements of long-term premorbid BP.

Second, our results reinforce the importance of BP control in midlife. We noted a significant latency effect of BP on SVD burden, especially for DBP, such that the associations with SVD burden were greatest with DBP measurements taken 10 to 20 years before TIA/ischemic stroke, or when readings were measured when patients were aged ≤55 years. In general, BP guidelines are primarily based on results from randomized controlled trials that have studied relatively short-term risks of cardiovascular events rather than the longer-term risk of clinical manifestations of cerebral SVD, such as vascular cognitive impairment or gait disturbances, which can be equally disabling. With trial follow-up of generally <5 years, the benefits of lowering BP in midlife in reducing the long-term consequences of SVD may have been substantially underestimated. For example, in the recent SPRINT (Systolic Blood Pressure Intervention Trial) which included a population with mean age of 68 years, followed-up for a median of 3.26 years, the absolute risk reduction for stroke in patients randomized to intensive BP lowering (SBP target <120 mm Hg) versus standard treatment (SBP target <140 mm Hg) was 0.06% per year, corresponding to a number needed to treat of 1667.^27^ It is possible that the number needed to treat to prevent the long-term consequences of SVD with BP lowering, during midlife would be much lower.

Third, our results demonstrate the importance of studying the age-specific associations between hypertension and SVD. As we age, our arteries stiffen and this is accompanied by a reduction in DBP.^11^ Analysing all patients together without stratification by age may therefore undermine potential strong age-specific associations of DBP with SVD. Although it remains unclear whether SBP or DBP is more important in the pathogenesis of SVD,^1^ we were able to demonstrate that the associations between mean DBP and SVD is most significant in younger individuals or when DBP is measured at younger ages. In contrast, mean SBP seemed to be significantly associated with SVD burden in the young and the elderly.

Although we consider our results valid, our study has several limitations. First, premorbid measurements of BPs were obtained in a retrospective manner from life-long primary care records. Without doubt, there would have been inconsistencies with regards to the measurement of BPs from practice to practice and from visit to visit, but any resulting inaccuracy would be expected to dilute any risk associations, suggesting that our findings are likely to be conservative. Our results are also consistent with a recent prospective cohort study with standardized BP measurements.^14^ Second, we used the total SVD score to represent the global SVD burden. Although the total SVD score was independently derived,^17^ has been shown to predict recurrent stroke,^20^ and it encapsulates a wide range of neuroimaging markers of SVD, more widespread validation in other studies would be helpful. Moreover, whereas other studies have tended to focus on the role of BP in individual imaging markers of SVD,^28–31^ individual markers may not fully capture the clinical significance of cerebral SVD. Third, patients were scanned on 4 different scanners during the study period. Although this may have been a potential source of heterogeneity, the mean total SVD scores were similar among patients scanned across the 4 scanners, and the prognostic value of the total SVD score was robust to scanner type and strength.^20^ Moreover, the risk associations between mean premorbid SBP with an increasing total SVD score was similar for all 4 scanners with no significant heterogeneity (Table V in the online-only Data Supplement). Finally, our analysis was limited to patients predominantly with a hypertensive form of SVD with relatively small numbers of patients with cerebral amyloid angiopathy (72 patients with strictly lobar microbleeds). Nevertheless, within this subset of patients, both mean premorbid SBP and DBP remained very strong predictors of an increasing total SVD score (age and sex-adjusted odds ratio of top versus bottom quartile of SBP: 24.78; 4.22–145.57; *P*=0.0004; DBP: 9.42; 2.50–35.45; *P*=0.001). However, these observations would need to be confirmed in larger cohorts.

## Acknowledgments

We are grateful to all the staff in the general practices that collaborated in the Oxford Vascular Study: Abingdon Surgery, Stert St, Abingdon; Malthouse Surgery, Abingdon; Marcham Road Family Health Centre, Abingdon; The Health Centre, Berinsfield; Key Medical Practice; Kidlington; 19 Beaumont St, Oxford; East Oxford Health Centre, Oxford; Church Street Practice, Wantage. We also acknowledge the use of the facilities of the Acute Vascular Imaging Centre, Oxford. K.K. Lau collected imaging data, did the statistical analysis and interpretation, wrote and revised the article. Drs Li and Simoni collected data. Z. Mehta did the statistical analysis. Dr Küker provided study imaging supervision. Dr Rothwell conceived and designed the overall study, provided study supervision and funding, acquired, analyzed and interpreted data, and wrote and revised the article.

## Sources of Funding

The Oxford Vascular Study is funded by the National Institute for Health Research (NIHR) Oxford Biomedical Research Centre, Wellcome Trust, Wolfson Foundation, British Heart Foundation, and the European Union’s Horizon 2020 program (grant 666881, SVDs@target). Dr Rothwell is in receipt of an NIHR Senior Investigator award. K.K. Lau is funded by a University of Oxford Croucher Scholarship. The view expressed are those of the author(s) and not necessarily those of the National Health Service, the NIHR, or the Department of Health.

## Disclosures

None.

## Supplementary Material
