# Supplementary material for: Long-Term Premorbid Blood Pressure and Cerebral Small Vessel Disease Burden on Imaging in Transient Ischemic Attack and Ischemic Stroke: Population-Based Study
Source: Stroke. 2018 Jul 25;49(9):2053–60. doi: 10.1161/STROKEAHA.118.021578 (PMC6116796; doi:10.1161/STROKEAHA.118.021578)
Supplement: Supplementary file 1 [file str-49-2053-s001.pdf]

SUPPLEMENTAL MATERIALS

Supplementary Tables

Supplementary Table I. Imaging sequence parameters of the study population

Supplementary Table II. Relationships of lacunes and Total Small Vessel Disease Score with baseline blood pressure, premorbid blood pressure (per SD increase) and history of hypertension

Supplementary Table III. Ordinal logistic regression analysis on the relationships of Total Small Vessel Disease Score with premorbid blood pressures measured (top vs. bottom quartile) within 1 year, 1-5 years, 5-10 years and 10-20 years of TIA/ischaemic stroke in patients aged <60

Supplementary Table IV. Ordinal logistic regression analysis on the associations between Total Small Vessel Disease Score with mean premorbid blood pressure (top vs. bottom quartile) stratified by age of which premorbid blood pressure measurement was recorded (odds ratios of premorbid blood pressure taken as top vs. bottom quartile as referent)

Supplementary Table V. Ordinal logistic regression analysis on the relationships of Total Small Vessel Disease Score with mean premorbid systolic blood pressure (top vs. bottom quartile), stratified by MRI scanner

Supplementary Table I. Imaging sequence parameters of the study population

MR parameters	OXVASC scanner 1<br>Magnetom Verio, Siemens Healthcare	OXVASC scanner 2<br>Discovery MR750, GE Healthcare	OXVASC scanner 3<br>Achieva, Philips Healthcare	OXVASC scanner 4<br>Signa HDxt, GE Healthcare
**Patients scanned**	375	60	481	93
**Field strength (T)**	3	3	1.5	1.5
**T1W TR/TE/TI (ms)**	2000/1.94/880	-	701/16	-
**T2W TR/TE (ms)**	6000/96	5800/94	5061/100	3760/100
**FLAIR TR/TE/TI (ms) (3D)**	9000/88/2500	9600/130/2350	11000/140/2800	8080/112/2200
**Diffusion TR/TE (ms)**	5300/91	6000/84	2891/73	6100/71
**GRE / SWI TR/TE (ms) (3D)**	GRE 504/15	GRE 500/20	GRE 694/23	GRE 560/25
**Pixel bandwidth (Hz)**	240 (T1W)<br>220 (T2W)<br>202 (FLAIR)<br>1374 (Diffusion)<br>200 (GRE)	-<br>50 (T2W)<br>41.7 (FLAIR)<br>250 (Diffusion)<br>31.3 (GRE)	87.4 (T1W)<br>88.5 (T2W)<br>375 (FLAIR)<br>25.3 (Diffusion)<br>109.3 (GRE)	-<br>47.6 (T2W)<br>31.3 (FLAIR)<br>-<br>75 (GRE)
**Matrix**	256x256 (T1W)<br>320x320 (T2W)<br>192x192 (FLAIR)<br>130x130 (Diffusion)<br>320x256 (GRE)	-<br>512 (T2W)<br>384x224 (FLAIR)<br>128x128 (Diffusion)<br>288x224 (GRE)	118x214 (T1W)<br>356x193 (T2W)<br>236x159 (FLAIR)<br>97x84 (Diffusion)<br>256x163 (GRE)	416x256 (T2W)<br>256x224 (FLAIR)<br>128x128 (Diffusion)<br>288x192 (GRE)
**No. of slices**	208 (T1W)<br>25 (T2W)<br>50 (FLAIR)<br>25 (Diffusion)<br>25 (GRE)	25	25 (T1W)<br>25 (T2W)<br>28 (FLAIR)<br>25 (Diffusion)<br>22 (GRE)	25
**Slice thickness (mm)**	1 (T1W)<br>5 (T2W)<br>3 (FLAIR)<br>5 (Diffusion)<br>5 (GRE)	5	5	5
**Inter-slice gap (mm)**	0 (T1W)<br>1 (T2W)<br>0 (FLAIR coronal)<br>1 (Diffusion)<br>1 (GRE)	1	1	1
**Voxel size (mm^3)**	1.0x1.0x1.0 (T1W)<br>0.8x0.8x5.0 (T2W)<br>1.0x1.0x3.0 (FLAIR)<br>1.8x1.8x5.0 (Diffusion)<br>0.9x0.8x5.0 (GRE)	-	0.53x0.53x5.0 (T1W)<br>0.65x0.65x5.0 (T2W)<br>0.82x0.81x5.0 (FLAIR)<br>1.74x1.73x5.0 (Diffusion)<br>0.90x0.90x5.0 (GRE)	-

Supplementary Table II. Relationships of lacunes and Total Small Vessel Disease Score with baseline blood pressure, premorbid blood pressure (per SD increase) and history of hypertension

	**Univariate OR (95% CI)**	**Age and sex adjusted OR (95% CI)**	**p**	**p (test of parallel lines)[‡]**
**Lacunes***				
Baseline systolic blood pressure	1.17 (0.99-1.39)	1.10 (0.92-1.30)	0.29	-
Baseline diastolic blood pressure	0.84 (0.71-0.99)	0.89 (0.75-1.06)	0.19	-
Hypertension	2.05 (1.45-2.89)	1.74 (1.22-2.49)	0.002	-
Mean premorbid systolic blood pressure	1.57 (1.33-1.85)	1.45 (1.21-1.73)	<0.001	-
Mean premorbid diastolic blood pressure	1.28 (1.10-1.49)	1.34 (1.14-1.57)	<0.001	-
**Total Small Vessel Disease Score[†]**				
Baseline systolic blood pressure	1.34 (1.19-1.51)	1.16 (1.03-1.32)	0.018	0.18
Baseline diastolic blood pressure	0.92 (0.82-1.03)	1.10 (0.98-1.25)	0.11	<0.001
Hypertension	2.53 (2.01-3.20)	1.61 (1.26-2.06)	0.0001	0.47
Mean premorbid systolic blood pressure	1.95 (1.73-2.19)	1.44 (1.27-1.63)	<0.001	0.054
Mean premorbid diastolic blood pressure	1.20 (1.08-1.34)	1.32 (1.18-1.49)	<0.001	0.45

OR=odds ratio; CI=confidence interval
*binary logistic regression; [†]ordinal logistic regression; [‡]based on age and sex adjusted model

Supplementary Table III. Ordinal logistic regression analysis on the relationships of Total Small Vessel Disease Score with premorbid blood pressures measured (top vs. bottom quartile) within 1year, 1-5 years, 5-10 years and 10-20 years of TIA/ischaemic stroke in patients aged <60

	Age <60 (n=251)		
	**Univariate OR (95% CI)**	**p**	**p (test of parallel lines)**
**Mean premorbid systolic blood pressure**			
Within 1 year	2.07 (0.78-5.47)	0.14	<0.001
1-5 years	2.32 (0.97-5.55)	0.060	<0.001
5-10 years	2.34 (0.82-6.68)	0.11	<0.001
10-20 years	3.65 (0.98-13.61)	0.054	0.97
**Mean premorbid diastolic blood pressure**			
Within 1 year	1.03 (0.38-2.80)	0.95	<0.001
1-5 years	0.96 (0.40-2.27)	0.92	<0.001
5-10 years	2.34 (0.90-6.08)	0.080	0.24
10-20 years	6.56 (2.47-17.41)	0.0002	0.79

OR=odds ratio; CI=confidence interval

Supplementary Table IV. Ordinal logistic regression analysis on the associations between Total Small Vessel Disease Score with mean premorbid blood pressure (top versus bottom quartile) stratified by age of which premorbid blood pressure measurement was recorded (odds ratios of premorbid blood pressure taken as top vs. bottom quartile as referent)

	Univariate OR (95% CI)	p	p (test of parallel lines)
**Systolic blood pressure**			
≤55 years	3.02 (1.46-6.27)	0.003	0.25
56-65 years	2.66 (1.68-4.19)	<0.0001	0.93
66-75 years	2.69 (1.71-4.22)	<0.0001	0.69
>75 years	2.34 (1.29-4.26)	0.005	0.58
**Diastolic blood pressure**			
≤55 years	3.14 (1.80-5.50)	<0.0001	0.41
56-65 years	2.31 (1.47-3.63)	0.0003	0.96
66-75 years	2.23 (1.43-3.50)	0.0004	0.05
>75 years	1.26 (0.70-2.27)	0.44	0.83

OR=odds ratio; CI=confidence interval

Supplementary Table V. Ordinal regression analysis on the relationships of Total Small Vessel Disease Score with mean premorbid systolic blood pressure (top vs. bottom quartile), stratified by MRI scanner

	Univariate OR (95% CI)	p
MRI scanner 1	4.70 (2.70-8.17)	<0.0001
MRI scanner 2	11.58 (2.83-47.23)	0.001
MRI scanner 3	9.16 (5.38-15.58)	<0.0001
MRI scanner 4	2.76 (0.94-8.05)	0.064

OR=odds ratio; CI=confidence interval
